# Extensive genetic admixture between Tai-Kadai-speaking people and their neighbours in the northeastern region of the Yungui Plateau inferred from genome-wide variations

**DOI:** 10.1186/s12864-023-09412-3

**Published:** 2023-06-12

**Authors:** Jiawen Wang, Jun Wu, Qiuxia Sun, Qian Wu, Youjing Li, Shuhan Duan, Lin Yang, Wenxin Wu, Zheng Wang, Yan Liu, Renkuan Tang, Junbao Yang, Chuanchao Wang, Chao Liu, Jianwei Xu, Mengge Wang, Guanglin He

**Affiliations:** 1 School of Forensic Medicine, Guizhou Medical University, Guiyang, 550004 China; 2 Institute of Rare Diseases, West China Hospital of Sichuan University, Sichuan University, Chengdu, 610000 China; 3 Department of Forensic Medicine, College of Basic Medicine, Chongqing Medical University, Chongqing, 400331 China; 4 Qiannan Prefecture People’s Hospital, Buyi and Miao Autonomous Prefecture of QianNan, Buyi and Miao Autonomous Prefecture of QianNan, 558000 China; 5 Congjiang People’s Hospital, Congjiang, 557499 China; 6 School of Basic Medical Sciences, North Sichuan Medical College, Nanchong, 637000 China; 7 Institute of Forensic Medicine, West China School of Basic Medical Sciences & Forensic Medicine, Sichuan University, Chengdu, 610041 China; 8 Department of Anthropology and Ethnology, School of Sociology and Anthropology, Institute of Anthropology, Xiamen University, Xiamen, 361000 China; 9 Faculty of Forensic Medicine, Zhongshan School of Medicine, Sun Yat-Sen University, Guangzhou, 510275 China; 10 Department of Pharmacology, School of Basic Medicine, Guizhou Medical University, Guiyang, 550004 China; 11 Center for Archaeological Science, Sichuan University, Chengdu, 610000 China

**Keywords:** Biological adaptation, Genome-wide SNPs, Genetic admixture model, TK people

## Abstract

**Background:**

Yungui Plateau in Southwest China is characterized by multi-language and multi-ethnic communities and is one of the regions with the wealthiest ethnolinguistic, cultural and genetic diversity in East Asia. There are numerous Tai-Kadai (TK)-speaking populations, but their detailed evolutionary history and biological adaptations are still unclear.

**Results:**

Here, we genotyped genome-wide SNP data of 77 unrelated TK-speaking Zhuang and Dong individuals from the Yungui Plateau and explored their detailed admixture history and adaptive features using clustering patterns, allele frequency differentiation and sharing haplotype patterns. TK-speaking Zhuang and Dong people in Guizhou are closely related to geographically close TK and Hmong-Mien (HM)-speaking populations. Besides, we identified that Guizhou TK-speaking people have a close genetic relationship with Austronesian (AN)-speaking Atayal and Paiwan people, which is supported by the common origin of the ancient Baiyue tribe. We additionally found subtle genetic differences among the newly studied TK people and previously reported Dais via the fine-scale genetic substructure analysis based on the shared haplotype chunks. Finally, we identified specific selection candidate signatures associated with several essential human immune systems and neurological disorders, which could provide evolutionary evidence for the allele frequency distribution pattern of genetic risk loci.

**Conclusions:**

Our comprehensive genetic characterization of TK people suggested the strong genetic affinity within TK groups and extensive gene flow with geographically close HM and Han people. We also provided genetic evidence that supported the common origin hypothesis of TK and AN people. The best-fitted admixture models further suggested that ancestral sources from northern millet farmers and southern inland and coastal people contributed to the formation of the gene pool of the Zhuang and Dong people.

**Supplementary Information:**

The online version contains supplementary material available at 10.1186/s12864-023-09412-3.

## Introduction

Tai-Kadai (TK) people are mainly distributed in South China and Southeast Asia, including Zhuang, Dong, Sui, Mulao, Maonan, Li, Gelao, Buyei and Dai, etc. According to historical records, the TK-speaking populations are descendants of the ancient Baiyue people from southeastern China [1]. Recent ancient mitochondrial DNA-based research speculated that the hanging coffin burial people might be the direct ancestors of the modern TK-speaking people [2]. Hanging coffins were popular among ancient southern Chinese people and Southeast Asians. It originated from the southeast coast of China, such as the Wuyi mountain area 3,600 years ago [2]. Historical and archaeological documents suggest that some ancient Baiyue people originated along the Yangtze River Basin and migrated to South China and Southeast Asia in the second century BC. Followingly the Han populations’ continuous southward expansion due to war and starvation further complicated the genetic landscape of ancient and modern people in South China. Thus, ancient northern East Asians were also viewed as one of the ancestors of modern TK-speaking people [3]. However, how population interaction between northern and southern East Asians influenced the pattern of genetic diversity of modern southern Chinese indigenes was kept unknown. In addition, haplogroup O1-M119, one essential patrilineal type of the TK-speaking populations, was found to have a high-frequency distribution in modern and ancient southern Chinese people near the Yangtze River Basin. It linked the rice farmers in the Yangtze River Basin with the contemporary TK ethnic groups [4]. Except for the findings drawn from the genetic studies, linguistic evidence also provided additional documentation of the historical background of the TK people. Linguistic research revealed a close relationship between the TK and Austronesian (AN) languages, and the two language families originated from and were dominant in South China. These groups were associated with the origin and expansion of rice farmers in ancient South China. According to archaeological discoveries, domesticated rice originated initially in Yuchunyan, Xianrendong and Shangshan sites along the Yangtze River Basin and was the main planting crop of the TK people. Wang et al. recently reported that the Yellow River millet farmers in the north genetically impacted all southern East Asians, including TK-speaking people [5]. A piece of genomic studies suggested that the early southward migration of the TK populations from South China may have impacted the diversity of the TK populations in Vietnam and Thailand [6–9]. Compared to Southeast Asia, TK people in South China had higher language diversity and several historical and linguistic documents recorded the origins of Proto-TK people in South China. This region is considered the origin of the TK-speaking populations associated with ancient rice farming development [10]. Therefore, the complex genetic structure and gene flow events of the TK populations need to be comprehensively explored to rebuild the entire landscape of the evolutionary and adaptative history of TK people in East Asia.

Yungui Plateau, also called the Yunnan-Guizhou Plateau, is located in the flatter highland areas or the mountainous area of rolling hills, gorges, and karst topography region of Southwest China, which stretches from Yunnan’s Red River Fault in the southwest, across most of Guizhou Province and to the Hunan’s Wuling Mountains in the northeast and neighbours Chongqing and Sichuan in the north and Guangxi in the South. TK people are widely distributed in the Yungui Plateau and the surrounding South China and Mainland Southeast Asia regions. There are still some debates on the admixture events and evolutionary history of the genetic diversity of the TK populations [7, 9, 11, 12]. However, what could be confirmed is that the complex genetic structure pattern of the TK people is caused by both cultural transmission (language browning) and demic diffusion (population migration and admixture) [13–15]. Genetic studies focused on the TK people from inland Southeast Asia found that the diversity of the TK-speaking groups in South China was the highest. Based on the historical, linguistic and cultural documents and recent genetic studies, it is consistently stated that Proto-TK-speaking people originated from South China and consistently southward migrated to Southeast Asia [6, 9]. Our previous genome-wide SNP-based research suggested the genetic structure of the Hlai people was a mixture of proto-TK and Han populations in inland East Asia, which was recently evidenced via the high-coverage genomes [16–18]. Wang et al. further revealed the close genetic origin between TK- and AN-speaking populations at the whole genome level, and both might descend from the rice farmers in the Yangtze River valley [5]. The TK language groups are widely distributed in South China and have become essential in Southeast Asia. Further genetic exploration of ethnolinguistically diverse TK people may alleviate the uncertainty brought by the widely different geographical locations for the accurate inference of the origin and adaptation of TK populations.

Anatomically modern humans originated in Africa and interbred with archaic Neanderthal and Denisovan people in Eurasia [19]. About 120,000–80,000 years ago, early modern people spread eastward along the “southern route” and reached southern East Asia. Humans expanded globally and adapted to the local specific environments of the arctic, cold and highland environment, dietary subsistence and parasite exposures [20]. The research shows that with the increase of latitude, the body fat percentage of the TK men decreases, the muscle mass increases (mainly the muscles of limbs), and the body fat percentage of women increases, and the muscle mass decreases (primarily the muscles of upper limbs and trunk decrease) [21]. However, the underrepresentation of ethnolinguistically diverse Chinese populations in human genomic research hindered the full discovery and understanding of the pattern of human genetic diversity and the function of human genetic variants [22, 23]. The lack of a population-specific genomic database may not only exacerbate human health inequalities and hinder personalized medical diagnosis, treatment and prevention of clinical disorders but also limit the exploration of the full landscape of origin, migration, admixture and evolution of unsampled modern worldwide populations, especially in Africa, Oceania and some regions of Asia [22, 24]. TK people are typical migratory and mixed populations in South China and Southeast Asia. However, their genetic diversity, population history and adaptative features were limited and should be investigated in the genomic cohorts.

Based on previous genetic, linguistic, ethnic, and archaeological findings, we have the basic knowledge of the admixture history of TK people from China and Southeast Asia. However, fine-scale genetic structure and adaptive history based on the allele frequency differentiation and sharing haplotype patterns of Guizhou Dong and Zhuang people from the Yungui Plateau keep unknown. Besides, the genetic relationship between Zhuang and Dong people and their genetic relationship with other populations must be explored. Therefore, here, we integrated high-density data of 679,920 SNPs from Zhuang and Dong groups in the Congjiang county from Guizhou Province with modern and ancient SNP data from worldwide or East Asian populations included in the Allen Ancient DNA Resource (AADR) version v54.1 [25, 26]. We also combined our data with high-coverage whole-genome sequencing (WGS) genomes from Human Genome Diversity Project (HGDP) and Oceania genomic resources [25, 26]. We conducted a comprehensive population genetic structure analysis using multiple statistical approaches, including principal component analysis (PCA), model-based ADMIXTURE, Fst, *f*_3_/*f*_4_ statistics, etc., to deeply analyze the genetic origin of the TK population in Southwest China and explore and determine the positive natural selection signals of the TK populations.

## Results

### Overall patterns of genetic structure inferred from descriptive methods

**Fig. 1:**
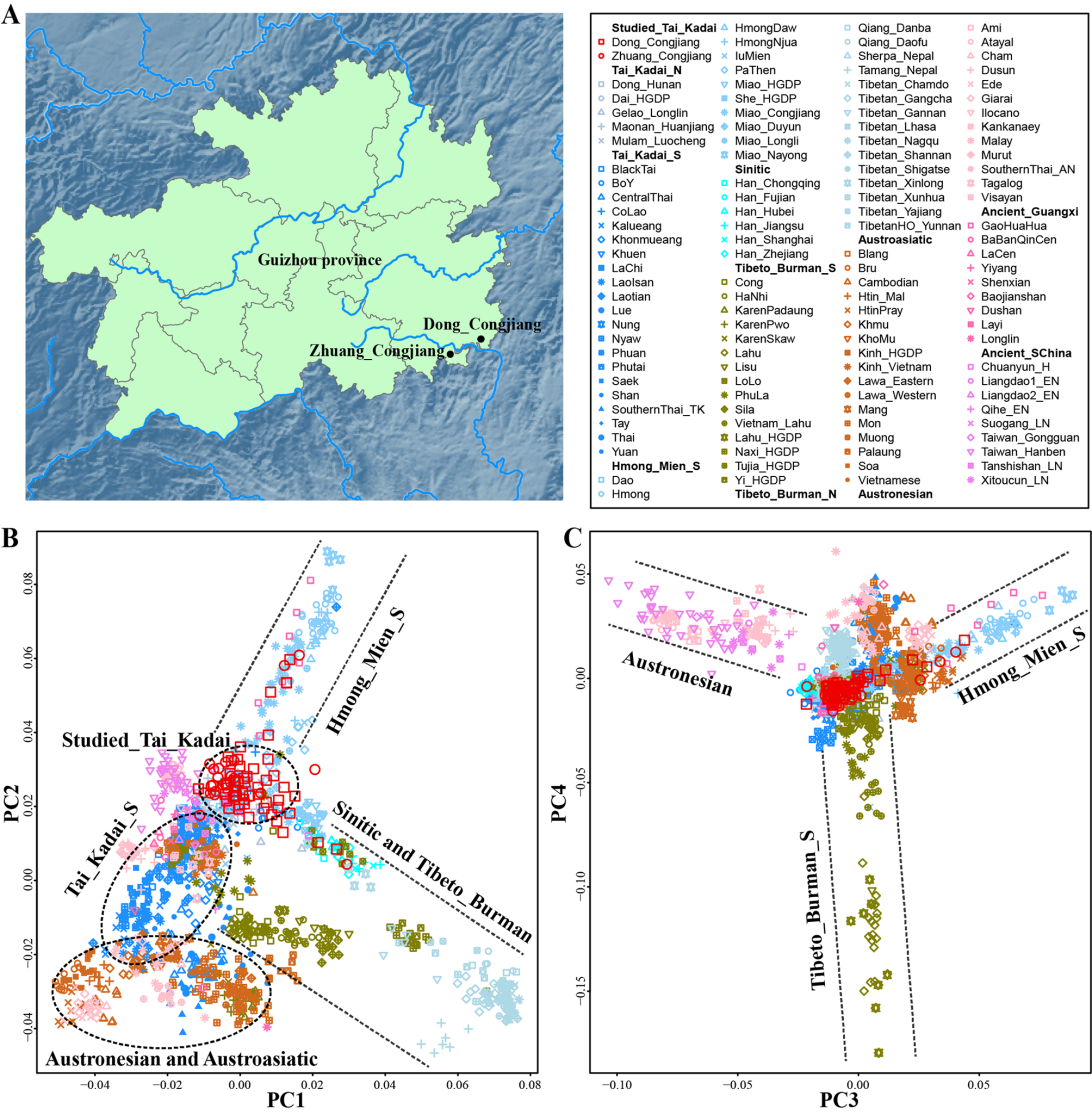
**General information of studied Guizhou TK speakers**. (**A**) The geographic positions of the two studied Guizhou TK populations in Southwest China. (**B ~ C**) Principal component analysis based on HO datasets and ancient East Asians (which were projected onto the essential background). Populations were shaped according to their unique ID from one language family. Different populations from the same language family or group were coloured

We generated genome-wide SNP data of 77 individuals from Zhuang_Congjiang (CJZ) and Dong_Congjiang (CJD) in Guizhou Province (Fig. 1A). We merged it with publicly available population data included in the AADR dataset, which is referred to as the merged HO dataset. We also called all SNPs from 929 whole genomes from HGDP [25] and 317 high-deep genomes from Oceanian genomic resources [26] and formed the 670 K WGS dataset (merged 670 K dataset). Firstly we explored the general population structure based on merged HO datasets using PCA and model-based unsupervised ADMIXTURE. Based on PC1 (0.74%) and PC2 (0.67%), we observed that HM, Sinitic and Tibeto_Burman (TB) speakers formed two main branches. The studied TK speakers, TK_S speakers, AN and Austroasiatic (AA) formed clusters respectively, but the studied TK overlapped with some populations of HM, TK_S, TK_N, Sinitic and TB populations (Fig. 1B). AN, HM and TB were distinguished and aggregated to form branches in PC3 (0.42%) and PC4 (0.34%) (Fig. 1C). PC1, PC2, PC3 and PC4 showed that the studied TK speakers formed a cluster and closed with HM, TK_S, TK_N, AN, Sinitic and TB populations (Fig. 1B **~ C**). When we projected ancient samples from Guangxi and South China, the studied TK populations overlapped with ancient populations from South China, including Taiwan_Hanben, Liangdao1_EN and Xitoucun_LN in PC1 (0.74%) and PC2 (0.67%) (Fig. 1B). However, according to the differentiation based on the variations extracted from the PC3 (0.42%) and PC4 (0.34%), we found that the studied TK populations had a close genetic relationship with most of the ancient_Guangxi populations (GaoHuaHua, BaBanQinCen, LaCen and ShenXian) (Fig. 1C).

**Fig. 2:**
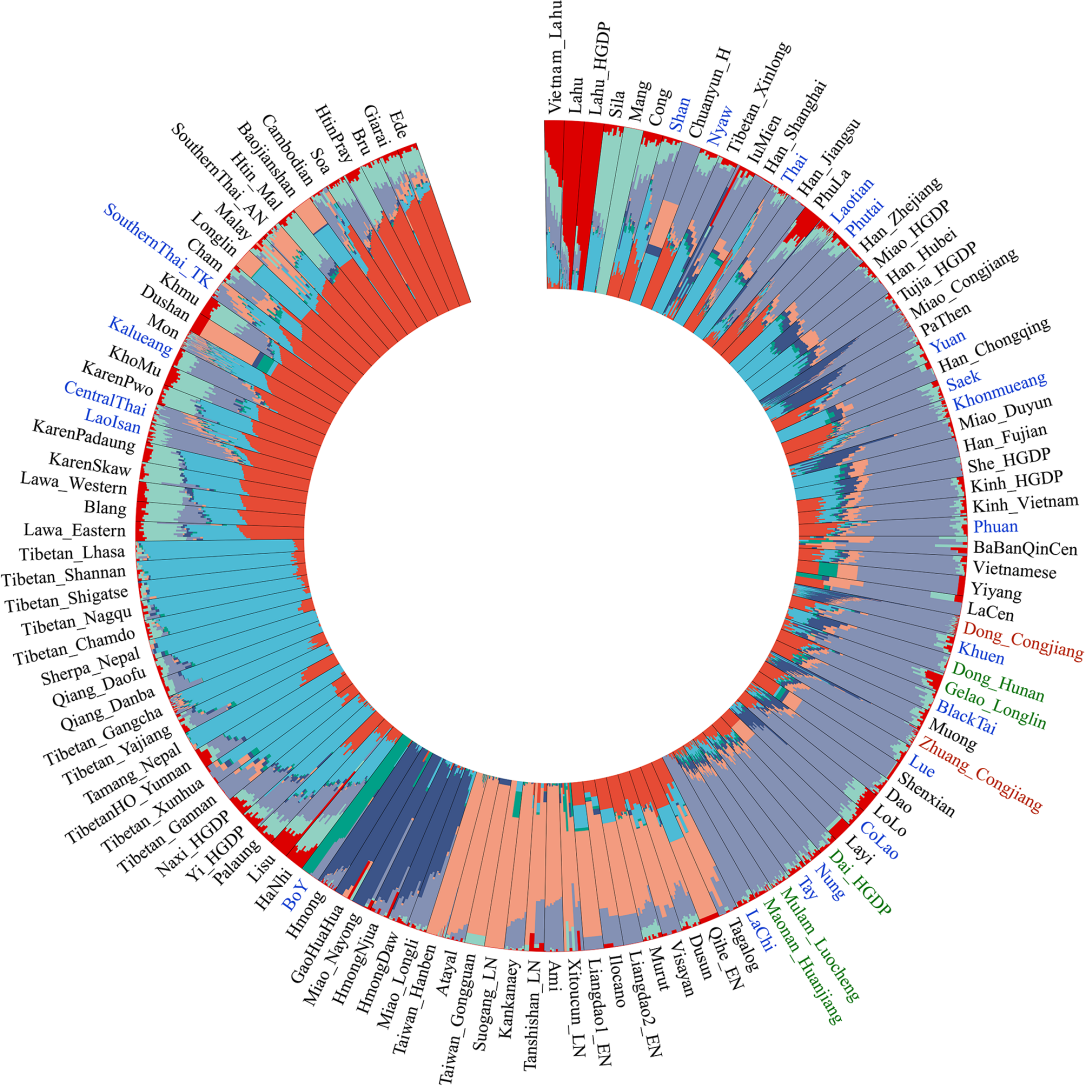
**Results of model-based ADMIXTURE clustering analysis**. Clustering patterns were visualized with the predefined ancestral sources at K = 8. All of these ancestral components were revealed in different colours

Next, we dissected and presented the ancestry proportions of CJZ and CJD using model-based ADMIXTURE in the context of modern and ancient reference populations, where the cross-validation error of the K = 8 model was the lowest (Fig. 2). The newly-genotyped TK populations have similar patterns of the fitted ancestral source and corresponding admixture proportions. The studied_TK populations harboured eight main types of ancestries (bright red, light green, gray, orange, dark blue, dark green, light blue, and dark red). The gray ancestry accounted for the highest proportion in studied TK populations and maximized in LaChi. Dark blue ancestry was enriched in modern HM populations (Hmong, Miao_Nayong, HmongDaw, HmongNjua and Miao_Longlin) and ancient Guangxi people (GaoHuaHua). The light blue ancestry was maximized in TB populations. The orange ancestry was enriched in modern AN populations and ancient South China populations. Moreover, the bright red, dark green and dark red ancestry composition of the studied TK populations respectively maximized in Lahu, BoY and AN populations (Fig. 2). In addition, in the ADMIXTURE model based on the merged 670 K dataset, we observed that the newly-genotyped TK populations harboured four main types of ancestries (red-brown, bright blue, light green and yellow) (Figure S1). In contrast, Dai people, who were also reported to be a TK-speaking population residing in the southwestern Yungui Plateau, possessed an ancestry composition that differed from theirs. ADMIXTURE results among East Asians showed that two predefined ancestral sources were well-fitted for the gene pool of studied populations (Figure S2). Our newly-studied TK populations comprised two ancestral components (orange and blue). The orange ancestral component was maximized in ancient people from the Yellow River, West Liao River, Wuzhuangguoliang and northern East Asia Coastal. The blue component was maximized in Hanben and Southern East Asia Island people.

### Fine-scale population structure inferred from the sharing haplotype patterns

**Fig. 3:**
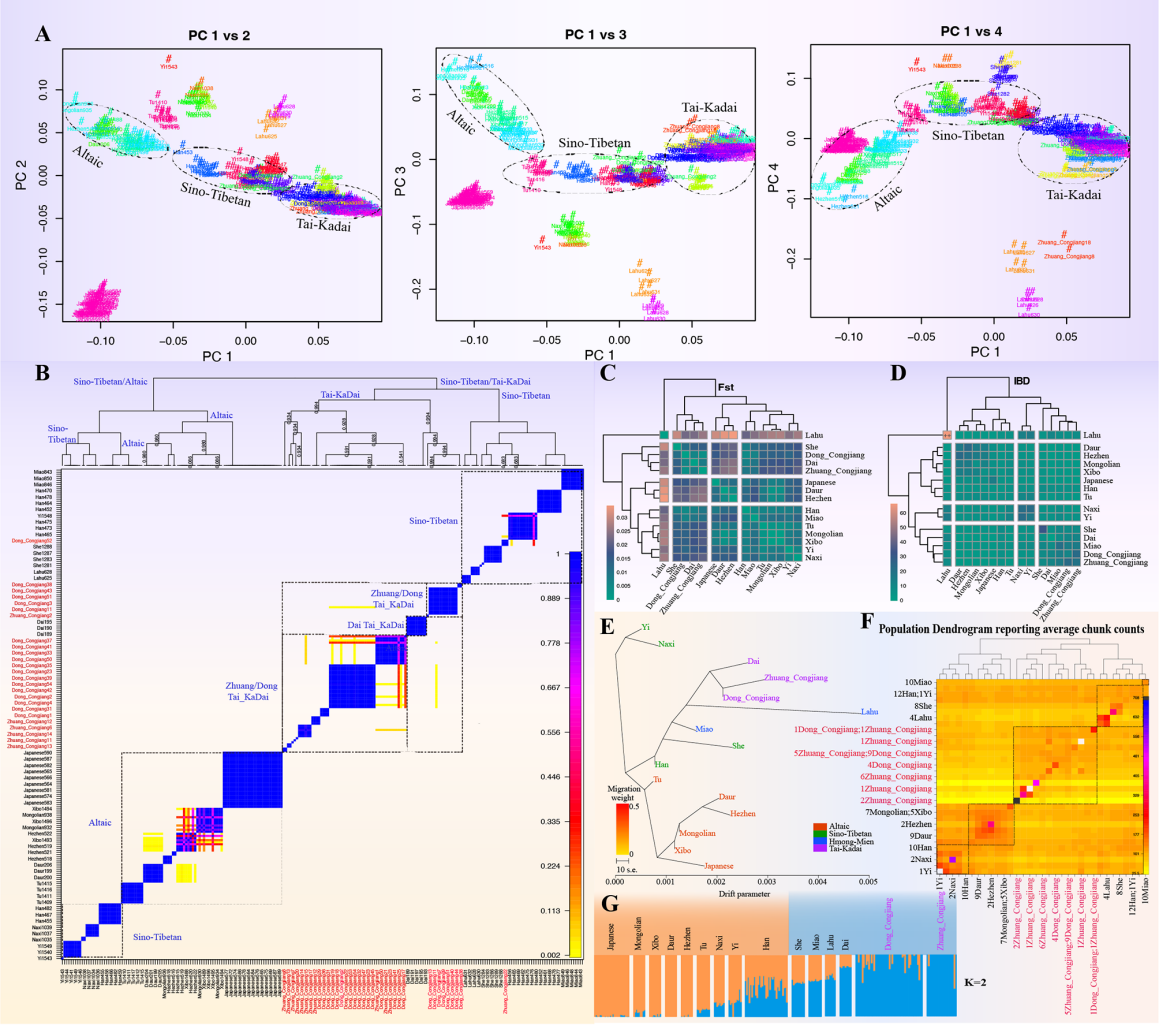
**Shared ancestry between TK speakers and their adjoining populations from China inferred from haplotype chunks**. (**A**) Two-dimensional plots of analyzed individuals based on their shared ancestry fragments. The different colours indicated the newly re-assigned population groups based on the newly identified homogeneous populations, mainly in line with the geographical and language origin. (**B**) Pairwise coincidence based on the co-ancestry coefficient based on the ChromoPainter-based shared haplotype patterns. (**C**) Pairwise genetic distance among 15 populations shared four genetic branches. (**D**) Average shared population-level IBD fragments showed four branches among 15 included populations. (**E**) TreeMix result of haplotype-analyzed datasets. (**F**) Population dendrogram based on the average chunk counts of genetically homogeneous populations. (**G**) Model-based ADMIXTURE among Chinese populations. Reference populations: HM groups (Miao), ST groups (Lahu, She, Daur, Han, Tu, Yi, Naxi), Altaic-speaking groups (Xibo, Mongolian, Hezhen) and TK groups (Dai)

To further dissect the genetic similarities and differences between newly-studied Guizhou TK populations and modern populations in East Asia, we explored the fine-scale population structure based on the genome-wide patterns of shared haplotype chunks generated from the merged 670 K dataset. We phased the haplotype fragments of 77 people from TK language families and other East Asians to explore the fine-scale population structure based on shared haplotype chunks (Fig. 3A). The newly-studied TK and Dai were clustered together, estranged from the Altaic-speaking groups in northern East Asia, and the geographically close HM and ST groups lay between them. Patterns of shared ancestry inferred from this population set confirmed the genetic clusters. The heatmap of the individual pairwise coincidence showed two main branches and fine-scale subbranches in line with their geographic or ethnic origins (Fig. 3B). The newly-studied TK populations shared the most ancestry chunks with Dais, followed by ST-speaking populations. To confirm the genetic affinity and population stratification, we calculated the pairwise Fst distance among them by analyzing the genetic differentiation index of newly-genotyped TK and surroundings populations (Table S1). We found that CJZ and CJD populations were more closely related to the Dai population (Fig. 3C) than other reference populations. Population comparisons also showed that CJZ and CJD populations were closely associated with geographically close HM and ST populations than other southern Chinese populations based on Fst genetic differences, such as the Han populations (0.0091 and 0.0048) and Miao (0.01 and 0.0065). Pairwise IBD sharing patterns also showed a close genetic relationship between the newly-studied TK and geographically close Miao and TK-speaking Dai (Fig. 3D). At the population level, paired coincidence matrix and TreeMix phylogenetic relationship revealed a clustering of TK-speaking populations, which followingly clustered on a branch with the geographically closer HM and ST populations (Fig. 3E~F). In addition, it was further shown that there was an inevitable genetic heterogeneity within modern TK-speaking groups according to ADMIXTURE results within East Asian groups (Fig. 3G).

To further explore the population substructure between Guizhou TK people and reference TK people from geographically different China and Mainland Southeast Asia, we merged our data with 60 publicly available TK-speaking populations and neighbours [6, 9, 27]. We conducted population genetic analysis based on allele and haplotyped approaches. The results of PCA showed that the newly-studied TK people overlapped with part of TK (Dong_Hunan, CoLao, BoY), HM (Miao_Congjiang, Miao_Duyun, She_HGDP), and Han_Hubei, which meant that the studied TK populations closed with TK, HM and Sinitic populations (Figure S3A). To explore the genetic heterogeneity and analyze the ancestral composition among TK populations, we conducted the model-based ADMIXTURE analysis and found that the admixture model with four ancestral components possessed the lowest cross-validation error (K = 4). Dark blue ancestry accounted for the largest proportion of the newly-genotyped TK populations, which was maximized in the AN (Atayal) people. Light blue and red ancestries enriched in the reported TK populations and geographically close HM populations, respectively. Green accounted for a smaller proportion, enriched in ST populations (Figure S3B). We found that CJD and CJZ firstly clustered in one cluster with geographically close HM (Miao_Duyun, Miao_Congjiang, Miao_Longli, Miao_Nayong) and then clustered with Mulam_Luocheng, Dong_Hunan and Gelao_Longlin in the TreeMix analysis (Figure S3C). Furthermore, CJD and CJZ were more closely related to geographically close Miao_Duyun (0.003 and 0.0004) and Miao_Congjiang (0.005 and 0.002) based on Fst genetic distance (Figure S3D). In addition, according to the heatmap of the individual pairwise ancestry coincidence, we observed that the newly-studied TK people and Miao_Duyun, Miao_Congjiang, Miao_HGDP, Dong_Hunan, CoLao formed a branch (Figure S3E). These results suggested that the newly-studied TKs from the northeastern region of the Yungui plateau possessed a differentiated genetic structure from other TK people (except Dong_Hunan, CoLao and BoY). These observed patterns of genetic diversity suggested that including ethnically diverse populations from China and Southeast Asia is essential for human genetic research, molecular anthropology and population-specific database construction for precision genomic medicine.

### Genetic affinity and admixture signatures inferred from the shared genetic drift

**Fig. 4:**
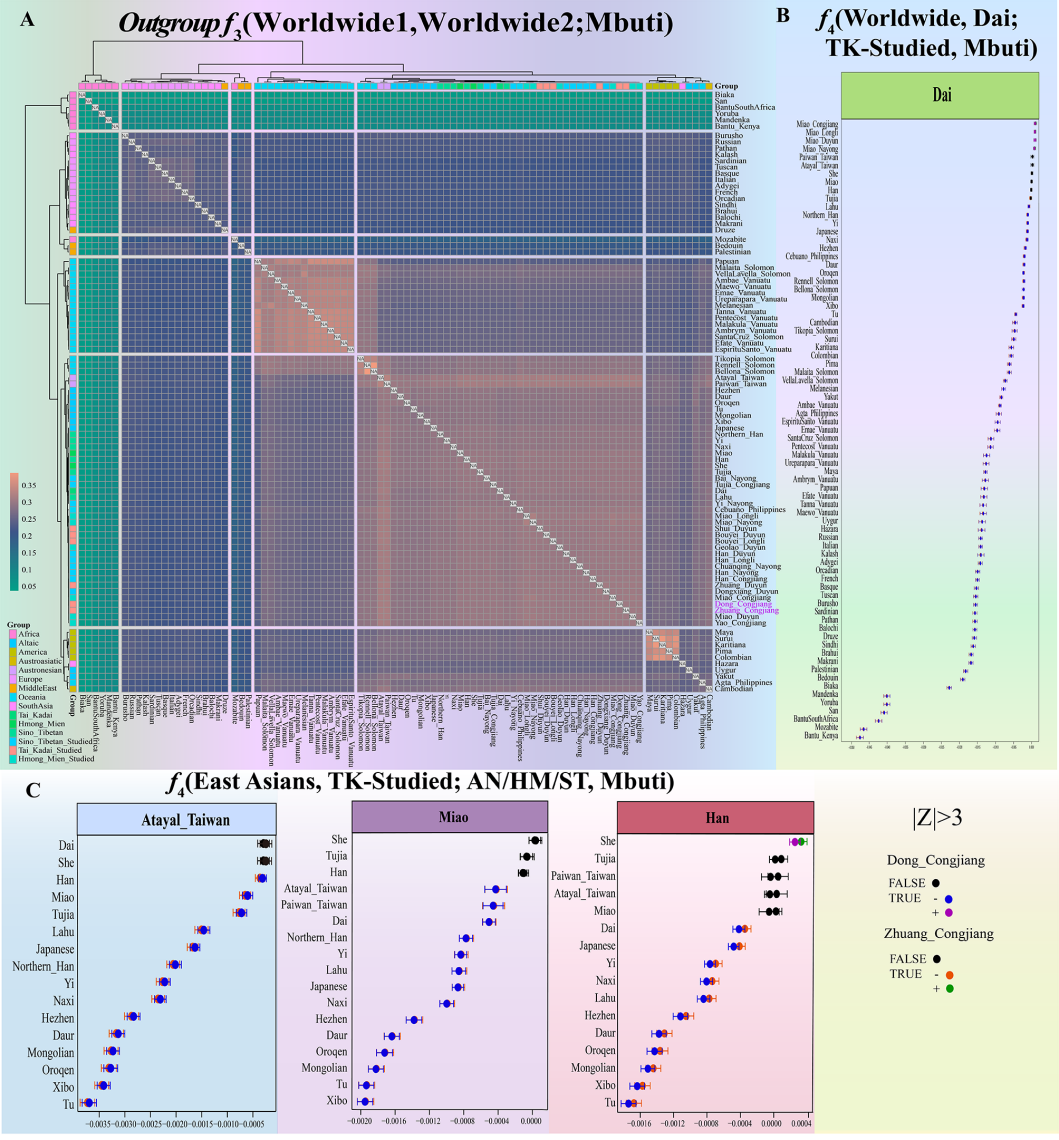
**Quantitative measurement for pairwise genetic affinity based on allele sharing**. (**A**) Outgroup-*f*_*3*_ in the form *f*_*3*_(X, Y; Mbuti) measured genetic drift between pairwise modern worldwide populations. Green indicates greater affinity and red indicates lower affinity among the pairwise populations. (**B**) The ancestry differences of the TK-speaking populations inferred from *f*_4_(studied TK, Dai; Reference, Mbuti). (**C**) *f*_4_(East Asian Reference, TK-speaking; AN/HM/ST, Mbuti) shows that compared with the northern East Asia populations, AN/HM/ST and TK-speaking shared more alleles. Atayal, Miao, and Han represent AN, HM, and ST populations. The true means that the Z-score is statistically significant, and the false means that it is statistically insignificant. Green and purple indicate that the Z-score of TK-speaking populations is greater than 3; Orange and blue indicate that the Z-score of TK-speaking populations is less than − 3; the black circle indicates Z-score − 3 ~ 3. The error bar is marked as the standard deviation

Additionally, to evaluate which contemporary groups shared more genetic drift with newly-studied TK populations, we conducted outgroup-*f*_3_-statistics of the form *f*_3_(X, Dong/Zhuang; Mbuti). Our findings demonstrated that the identified clustering pattern based on the outgroup-*f*_3_ matrix was compatible with intercontinental geographic division (Fig. 4A). When we focused on the shared drift between newly-studied and East Asian populations, we found that the studied TK populations generally had excessively shared alleles with AN-speaking Paiwan and Atayal from Taiwan Island based on the shared highest outgroup-*f*_*3*_ vales (Fig. 4A and Table S2). We also found that other southern Chinese populations shared more genetic drift than northern Chinese populations, such as Altaic people, and some genetic differences between geographically different TK people. These patterns of the observed genetic affinity were consistent with the documented affinity between Austronesian and Hainan TK people and differentiated admixture landscapes with neighbouring populations [16]. The estimated allele-sharing profiles revealed by *f*_3_-statistics have identified ancestral sources highly associated with northern and southern East Asians. We performed a series of *f*_4_-statistics to test the excess allele sharing with any representative sources formally. We first carried out symmetrical *f*_4_(CJD, CJZ; Reference populations, Mbuti) to explore the genomic heterogeneity or homogeneity among all studied TK populations (Table S3). The results showed that the CJZ shared more alleles with Dai than with the CJD. All other tested *f*-statistics in this form showed no statistically significant values, suggesting Zhuang and Dong people were relatively homogeneous compared with other East Asian reference populations. The significantly positive Z-scores of *f*_4_(Reference HM populations, Dai; TK-speaking people, Mbuti) suggested that the studied TK-speaking people shared more alleles with the geographically close HM populations than Dai (Fig. 4B). Additionally, the Guizhou TK-speaking people and Dai shared more alleles compared to non-East Asian and northern East Asian groups, consistent with the relative genetic homogeneity within southern Chinese populations compared with genetically or geographically distant reference populations (Table S4). Interestingly, we did not discover any statistically significant negative Z-scores in the form of *f*_4_(AN, Dai; TK-speaking people, Mbuti) (Fig. 4B), which showed that TK-speaking people did not share more alleles with Dai than AN. However, based on a Treemix analysis, including the modern AN groups, we observed that the TK-speaking people formed a clade with Dai (Figure S4). The considerably negative values of *f*_4_(East Asians, TK-speaking people; Atayal_Taiwan, Mbuti) further reinforced the findings that AN-speaking populations in South China shared more alleles with the TK-speaking people compared to northern East Asians (Fig. 4C). Similar to this observed pattern, Miao and Han shared more alleles with TK people than Altaic-speaking people in north East Asia (Fig. 4C).

In the ADMIXTURE model based on 6 Han populations and 7 TK populations, we observed that blue accounted for the much proportion in TK populations and riched in Han populations at K = 2 (Figure S5A). Based on the analysis of Fst genetic distance, we found that the genetic differentiation index of the TK and Han populations was less than 0.02. These results indicated that TK population may have obtained the gene flow of the Han population (Figure S5B). We further performed admixture *f*_3_-statistics in the form *f*_3_(source1, source2; TK-speaking groups) to explore the potential ancestral sources from 126 sampled modern and ancient Eurasian populations (Tables S5 ~ 6). The source pairs with statistically significant negative *f*_3_ values with Z-scores less than − 3 could be regarded as two ancestral source proximities explaining the allele frequency patterns observed in the tested populations. We identified 168 pairs that showed statistically negative *f*_3_-values in CJD, which denoted that the allele frequency of the targeted CJD intermediated between that in source1 and source2. The values of admixture *f*_3_-statistics tended to be exceptionally high when we used Daur, Xibo, Oroqen, Hezhen, Mongolian, Japanese, Tu, Oroqen and Northern_Han as the ancestral North East Asian sources and used HM-speaking populations as the ancestral South East Asian sources. The most apparent admixture signatures appeared in *f*_3_(HM, ST; CJD), suggesting that ancient people related to geographically close HM people and northern ST people contributed to the gene pool of Guizhou Dong. When we used ancient East Asian populations as primary ancestry of potential ancestral populations, we found an admixture signature when Shenxian and China_Miaozigou_MN were used as the source populations, such as *f*_3_(Shenxian, China_Miaozigou_MN; CJD) = − 3.153*SE. The Gaohuahua population from Guangxi also generated a robust mixed signal with northern East Asian populations, as shown by the fact that the Z-score of *f*_3_(Han, Gaohuahua; CJD) was − 6.668. However, we did not observe significantly negative Z-scores in admixture *f*_3_-statistics in the form of *f*_3_(source1, source2; CJZ), which showed that Zhuang people obtained relatively little recent gene flow from others (Table S6).

### Admixture landscape inferred from qpAdm, linkage disequilibrium decay and sharing haplotypes

We used qpAdm and ALDER programs to calculate the admixture proportions and admixture times. Firstly, we fitted two-way admixture models using the Yellow River millet farmers as the northern source and ancient people from Taiwan Island and Guangxi as southern ancestral sources to estimate the admixture proportions. We observed that the Yellow River millet farmers (50%) and Atayal_Taiwan (50%) both contributed to the gene pool of TK people (Table S7). When Yiyang was used as the southern source, it contributed around 80% of the ancestries to our targeted people, and the contribution of ancient inhabitants from the Yellow River valley accounted for roughly 20% (Table S7). Furthermore, we used southeastern East Asians (represented by Paiwan_Taiwan and indigenous AN-speaking Taiwanese), southern East Asians (represented by Dushan), and northern East Asians (represented by Shimao_LN) as three proxies of the possible ancestral sources to model the admixture proportions of the TK populations. Northern East Asian and southeast Asian ancestors contributed similar proportions to TK-speaking populations in the three-way admixture model (Table S8).

Secondly, we estimated the admixture date based on the exponential decay pattern of admixture-induced linkage disequilibrium and probability patterns of two chromosome ancestry segments in admixed individuals derived from two ancestry sources to explore when ancient populations from the northern and southern East Asians entered the gene pool of studied TK populations. Previous ancient genome research reported that the ancestors of TK-speaking people might be 1500-year-old BaBanQinCen, a possible ancestor who speaks TK languages, a meta-population of archaeological sites that lived in Guangxi 1,500 years ago [5]. Our results showed evidence of admixture for CJZ ~ 59.2±12.71 generations (1657.6 years) ago and CJD ~ 79.01±24.82 generations (2212.28 years) ago in the Miao_Nayong/Northern_Han model (Table S9), which suggested that the interaction between Miao and Han facilitated the formation of Zhuang and Dong in different time scales. Using the shared haplotype information, we further identified the ancestral sources and found that geographically close Miao and northern Han Chinese were the best-fitted ancestral source surrogates. Further, estimated admixture time and proportion focused on Dong showed Han Chinese (0.51) and Miao people (0.49) had extensive admixture seven generations ago. Further parameter estimation focused on Zhuang people showed that one-date with two ancestral sources was the best guess for the formation of Zhuang. Han contributed 0.27 ancestry and Miao contributed 0.73 ancestry to the gene pool of Zhuang people 17 generations ago.

### Biological adaptative signatures among TK-speaking populations

**Fig. 5:**
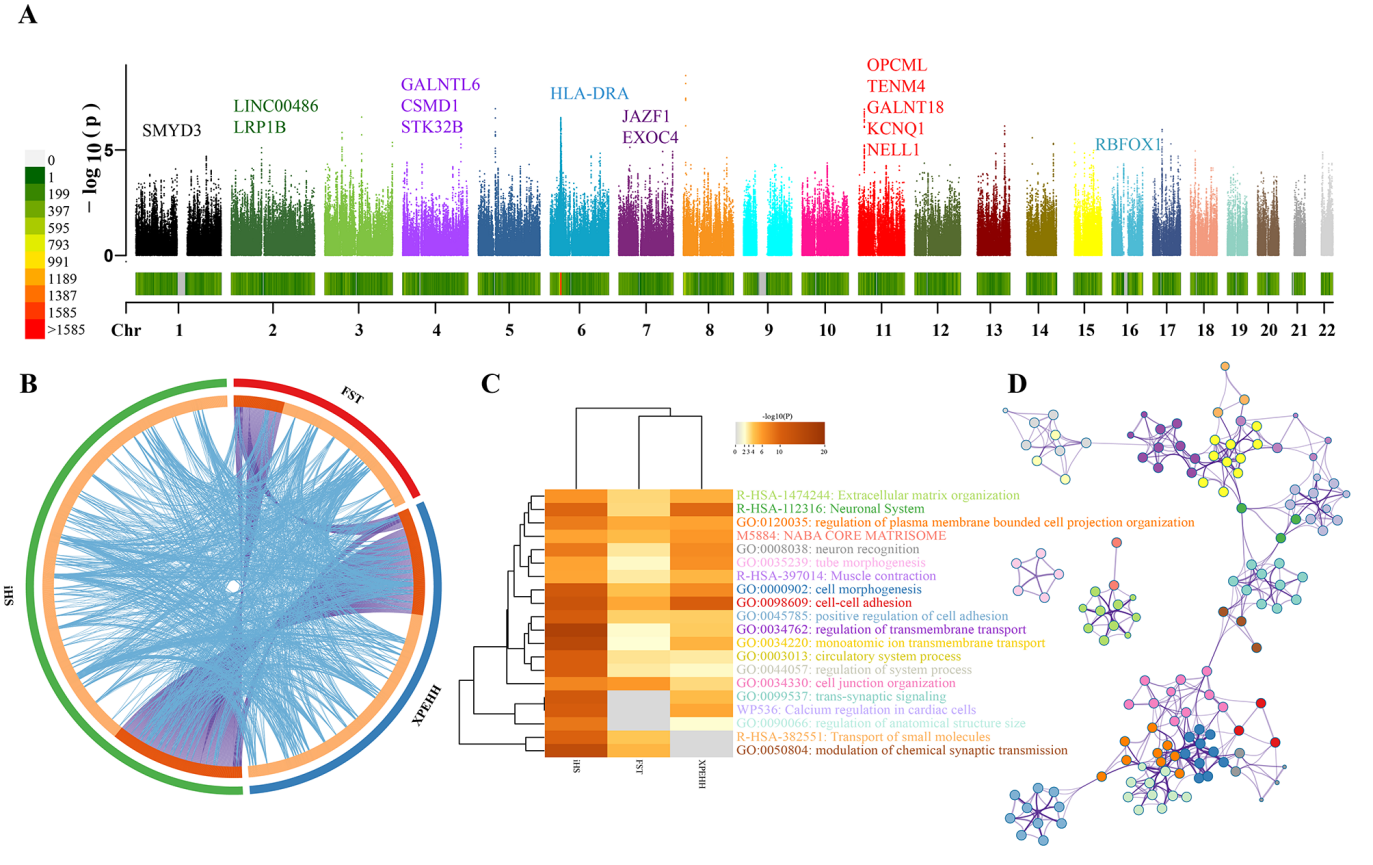
**Manhattan showed the natural selection signatures and enrichment analysis**. (**A**) p-values of XPEHH for Zhuang and Dong people using southern Hans as the reference population. Positive selection signals for genes screened for the intersection of Fst, XPEHH and iHS. (**B**) Overlap among three gene lists based on gene level and shared term level, where purple curves link genes that belong to the same enriched ontology term. (**C**) Top 20 of the GO-BP, KEGG, Reactome Gene Sets, WikiPathways and Canonical Pathways. (**D**) Network of enriched terms coloured by cluster-ID. The respective colour represents its cluster identity, and the size of a node is proportional to the number of input genes that fall into that term. Terms with a similarity score > 0.3 are linked by an edge (the thickness of the edge represents the similarity score)

Three different statistical methods were used for genome-wide scanning of the possible natural selection adaptative loci. We used the frequency-based genetic differentiation-based method (Fst) to identify the highly-differentiated variants and used haplotype-based approaches, including the cross-population extended haplotype homozygosity (XPEHH) and integrated haplotype score (iHS), to examine the status of sweep selection in TK people. Ultimately, we screened 15 genes based on the intersection obtained by XPEHH, iHS and Fst, namely *CSMD1*, *EXOC4*, *GALNT18*, *GALNTL6, HLA-DRA, JAZF1, KCNQ1, LINC00486, LRP1B, NELL1, OPCML, RBFOX1, SMYD3, STK32B* and *TENM4*. These targeted loci were used as the top selected adaptive genes, whose annotation information was mainly associated with the immune system and neurological disorders (Fig. 5A and Table S10). Some functions of these genes were also related to neurological disorders. *CSMD1*, CUB and Sushi multiple domains 1, were reported to be involved in many behavioral processes, such as memory and learning [28]. *EXOC4* is a gene related to neuron maintenance and neurotransmission [29]. *LRP1B* and *APP* combination reduce Aβ production, thus protecting the ageing process from cognitive dysfunction [30]. *OPCML* is a susceptibility gene of schizophrenia, which can regulate spinal maturation and cognitive behavior through Eph-Cofilin signal transduction [31]. *RBFOX1* regulates alternative splicing of tissue-specific exons and differentially spliced exons during erythropoiesis [32].

The association of *HLA-DR* genes with rheumatoid arthritis (RA) patients varies among different ethnic groups (or geographic regions) in China. DRB1*10 and DRB4*01 may be susceptibility genes for Zhuang RA patients. *HLA-DRA* acts as the sole alpha chain for *DRB1, DRB3, DRB4* and *DRB5* and plays a central role in the immune system and response by presenting peptides derived from extracellular proteins, particularly pathogen-derived peptides of T cells. Therefore, TK’s RA susceptibility may induce positive selection. Meanwhile, we also focused on the selection signals associated with the pathogenesis of diabetes, *JAZF1* and *KCNQ1*. *JAZF1* acts as a transcriptional corepressor of orphan nuclear receptor *NR2C2* and plays a role in glucose homeostasis by improving glucose metabolism and insulin sensitivity [33]. *KCNQ1* is associated with type 2 diabetes (T2D) [34].

Furthermore, we performed functional annotation and enrichment analysis for 329, 562 and 926 candidate genes screened based on Fst, XPEHH and iHS, respectively. The overlap between the three sets containing these genes and the pathways enriched to them was visualized (Fig. 5B). GO functional enrichment analysis revealed that natural selection genes were mainly enriched in several biological processes, such as regulating ion transport and modulation of chemical synaptic transmission. These biological processes played an important role in the pathways of the neuronal system and the transport of small molecules (Fig. 5C). The relationship between enriched pathways and biological functions was visualized, and we found that cell-cell adhesion and cell morphogenesis are closely related to the development of the nervous system brain (Fig. 5D).

## Discussion

The multi-ethnic and multi-lingual environment in Southwest China has created more possibilities for the original formation of the pattern of genetic diversity of the TK population in South China. Further genetic studies of TK populations in Southwest China will better define the genetic structure and evolutionary history of TK people. Therefore, we comprehensively integrated and analyzed the genome-wide SNP data of the CJZ and CJD from Guizhou with the publicly available reference population data, explored the fine-scale genetic structure of the TK populations, and illuminated its genetic relationship with ancient and modern reference people. In addition, in the fine-scale population structure, we found two samples with high outliers, which may be caused by a genetic mixture resulting from recent long-distance migration and were removed to ensure the accuracy of the analysis results. We also explored the natural selection signals of the CJZ and CJD based on different statistical approaches. Our results showed the gene pool of Guizhou TK people was formed via complex admixture processes inferred from the multiple best-fitted mixed models with northern and southern East Asians. We also found an inevitable genetic heterogeneity within the TK populations. In addition, we also discovered that Zhuang and Dong people were formed in different time scales due to the complex population interaction between northern and southern East Asian ancestral sources. Finally, we inferred the possible genetic signatures associated with the genetic susceptibility of some clinical diseases in the Guizhou TK populations based on the natural selection signals screened from genome-wide SNP data.

### The genetic affinity between TK people and southern East Asian populations and the North-South East Asian admixture model

We found that Guizhou TK populations had enriched genetic diversity and possessed strong genetic affinity with southern East Asians based on PCA and model-based ADMIXTURE analysis. The lower genetic differentiation indices within the studied CJZ and CJD populations and between them and Yunnan TK-speaking Dai suggested that the newly-genotyped TK populations have more substantial genetic similarity with those speaking similar languages [14, 35]. The genetic affinity between TK people and Guizhou HM and ST people also showed genetic connection and admixture between TK people and their geographically close neighbours. Linguistic evidence showed that the language components of the TK are highly similar to those of the HM and ST neighbours [36], suggesting that cultural diffusion was accompanied by human population movement and genetic admixture. Our previous genetic studies also illuminated the phenomena of population admixture and language browning between TB-speaking Tujia and Han Chinese [37]. Additionally, we also found that our studied TK populations shared more ancestral components with AN-speaking Atayal people collected from Oceanian genomic resources based on the four population tests and ADMIXTURE results [26]. The higher shared genetic drift based on the outgroup *f*_3_-statistics indicated that more alleles were shared between studied TK-speaking and AN-speaking people. Our results are consistent with previously reported genetic sharing patterns that supported a unique genetic link between AN-speaking and TK-speaking populations based on geographically different population data [16, 38]. Archaeological studies, cultural and linguistic documents have also suggested that the common ancestors of the TK and AN populations may have lived in coastal areas in southeastern China and subsequently migrated to the Taiwan Province and Southwest China in two subgroups [39].

The ancestral composition of Guizhou TK populations inferred from the two-way or three-way admixture models suggested that the primary ancestry of TK people derived from Yellow River millet farmers in northern East Asia and AN-speaking Taiwan indigenous people associated with ancient rice farmers. The fitted models indicated that the gene flow from ancient millet farmers in the Yellow River valley and rice farmers from the Yangtze River valley contributed to the formation of Guizhou TK populations. We found that the genetic diversity of CJD resulted from the genetic admixture of ancient and modern populations in southern and northern East Asia based on the admixture *f*_3_-statistics. This is consistent with the historical records that the ancient Baiyue people in South China migrated to the southwest and mixed with Hans and other indigenous populations to form the ancestors of TK populations. About 2,000 years ago, many northern Han Chinese migrated southward and arrived in southwestern China (Yunnan and Guizhou) to strengthen the dynasty’s rule and capture the Baiyue region [40]. Recent ancient DNA evidence also provided clues for the complex population movements and admixture between northern and southern East Asians and the population connection between inland and coastal southern East Asians [5, 27, 41]. Yang et al. reported Neolithic to historic genomes from Shandong and Fujian Provinces and found that Neolithic southward gene flow contributed to the formation of the gene pool of Fujian late Neolithic people from Tanshishan and Xitoucun sites [41]. Wang et al. reported Iron Age genomes from the Hanben site with a larger population size and identified additional gene flow from northern China associated with the millet agriculture dispersal, which influenced the gene pool of the ancestor of modern AN people. Recently, Wang et al. reconstructed the population transition from Holocene to historic Guangxi people and discovered the ancestry of modern TK people shared a similar genetic ancestry composition with historic Guangxi populations dating back around 1500–500 years ago, which also pointed to the southward gene flow from ancient Shandong and Henan people contributed to the formation of modern TK people [5]. ALDER-based results also identified the connection between northern and southern East Asia that occurred 1500 years ago. Haplotype-based admixture time estimation with different ancestral proximities showed the additional complex and later population connection and admixture from surrounding HM and ST people.

### Genetic heterogeneity within TK populations in Guizhou

Although the newly-studied TK populations had similar composition and close genetic relationship with previously reported TK populations (Dong_Hunan) and geographically close HM populations, we observed that the proportion of ancestral composition of reported TK populations (except Dong_Hunan) was completely different from that of CJZ and CJD. ADMIXTURE results showed that the TK-related component in reported TK was higher than that in CJZ and CJD. Previous studies have also revealed the genetic differentiation among TK populations from different regions, such as the Hainan island TK people and Chinese mainland TK people [16, 42, 43]. Genetic differentiation among geographically close TK people was also observed in TK people from Southeast Asia. A recent genetic study including TK populations from northern, northeastern, central, and southern Thailand revealed their different genetic admixture histories and differentiated genetic structures [9]. However, we have observed the effect of geographical location on genetic differences and also found that different ethnic groups from the same geographical area and the same language family trended to have genetic differences. Based on pairwise Fst distance, we found that the genetic relationship of CJD and CJZ was close to the geographically close HM populations. Additionally, we estimated the admixture signatures using the admixture *f*_3_(source1, source2; CJD) and identified the geographically close HM, modern and ancient northern East Asians contributed these mixed signals, especially with the Hans. This was consistent with the genetic patterns observed in PCA. In *f*_4_(TK-speaking1, TK-speaking2; reference populations, Mbuti), the CJZ shared more alleles with Dai compared with the CJD (Table S3). Interestingly, our results also showed that the time when Miao and Han mixed to form Zhuang and Dong seemed different. Therefore, further whole-genome sequencing is needed to deeply analyze the genetic structures of geographically diverse TK populations and eliminate their evolutionary history and genetic relationship with other ethnolinguistically diverse East Asian people.

### Biological adaptation may be an essential factor affecting the genetic diversity of TK populations

Genetic studies showed that ethnolinguistically diverse populations underwent different statuses of pathogen exposure, which may remain as different patterns of the allele frequency spectrum and extended haplotype homozygosity under natural selection processes. We explored the genome-wide candidate loci targeted by natural selections based on the reconstructed haplotype data and allele frequency differentiation. The *HLA-DR* alleles strongly associated with Chinese RA patients are *HLA-DRB*1*0405, 0401, 0404, 0410, etc. [44]. However, the association of *HLA-DR* genes with RA patients varied by ethnicity (or geography) [45]. The prevalence of RA is also higher in the Chinese TK population. *HLA-DR* (DRB1*10 and DRB4*01) has been shown to be a susceptibility gene for the development of RA in the TK population [46]. *HLA-DRA* genes play an essential role in immune diseases such as RA and SLE [47] and may be involved in the pathogenesis of immune system diseases in the TK population. The study on the association between *HLA-DRB1* haplotype and RA susceptibility in Han populations shows that the HLA-DRB1*0405 gene isassociated with RA in the Han population (*P* = 1.35 × 10^− 6^) [48]. Genetic traits resulting from natural selection differ among different ethnic groups and are also related to the natural environment of geographical location. The humid environment of Southwest China may contribute to the high incidence of RA in the TK populations. Many patients report that weather and season will affect their symptoms. It is found that the pain degree of rheumatoid arthritis is positively correlated with humidity, temperature and air pressure [49]. Additionally, we observed some genes are involved in the occurrence and development of diabetes. *JAZF1* is associated with assessing reduced glomerular filtration rate and is involved in the pathogenesis of T2D by regulating lipid metabolism processes. It has been shown that *JAZF1* is associated with T2D in the Chinese ST population (Han and She) [50, 51], which may also be involved in the pathogenesis of T2D in TK populations. Generally, our comprehensive population admixture modelling focused on Guizhou TK people have identified complex admixture pattern in southwestern China, which can provide important genetic evidence for further studies in linguistics, ethnology, archaeology, genomic medicine and population genetics and forensic science. We emphasized the importance of collecting more high-coverage WGS data from ethnolinguistically diverse Chinese populations and establishing a population-specific database for personalized precision medicine. As we all know that although international large-scale genomic studies, such as UK Biobank [52], Trans-Omics for Precision Medicine (TOPMed) [53] and the Genome Aggregation Database (gnomAD) [54], have achieved considerable advances for genomic medicine, the European bias in human genetic studies also has the possibility to introduce human inequality. Chinese genomics projects, including the China Metabolic Analytics Project (ChinaMAP) [55], 10K_CPGDP (Chinese Population Genetic Diversity), NyuWa Genome resource [56] and Westlake BioBank for Chinese (WBBC) [57] have tried to promote filling the gap of East Asian-specific population database. However, most newly-reported genetic variations and population genomic sources only focused on Han Chinese populations. To capture and understand the full landscape of Chinese population genetic variants and their medical relevance, we should include more high-depth genomes from ethnolinguistically diverse populations such as the high-altitude highlanders in the Tibetan Plateau and east-west admixture Altaic-speaking people in northwestern China.

## Conclusion

Taken together, our work focused on the genome-wide SNP data of TK populations from Southwest China, directly demonstrating their genetic affinity with ancient people in the Yellow River and Yangtze River Basins and suggesting the complex admixture processes that contributed to the formation of Guizhou TK people. We also identified Guizhou people who shared a close genetic relationship with geographically close HM people and geographically distant AN-speaking Atayal and Paiwan people, suggesting ancient genetic common origin and recent population admixture played an important role in the observed patterns of genetic diversity of modern TK people. Additionally, unique patterns of naturally-selected signatures in TKs have identified many candidate genes associated with biological processes and pathways of the crucial immune system. In general, our findings provided direct evidence that supported an admixture model in which TK populations are a mixture of North and South East Asian populations and complex interaction with geographically close HM people and Han Chinese.

## Materials and methods

### Sample collection and DNA extraction

We randomly collected 77 TK-speaking samples (23 Zhuangs and 54 Dongs) from Congjiang County in Guizhou Province, Southwest China. The parents and grandparents of these samples participating in this study were all indigenous people who had lived here for at least three generations and were not genetically related to each other. Genomic DNA was extracted and isolated by drawing 200ul of blood from each sample and diluting it with 30ul of genomic elution buffer using the PureLink Genomic DNA Mini Kit. Subsequently, we performed a preliminary determination of DNA concentration using a NanoDrop 2000 spectrophotometer. Second, the 7500 real-time PCR system was used to accurately quantitate DNA concentration using the Quantifiler Trio DNA Quantification Kit and the Quantifiler Human DNA Quantification Kit according to the appropriate instructions. 77 DNA samples were genotyped using the Affymetrix WeGene V1 array, which covered about 700 K SNPs. And SNPs with low-profile or batch effects were removed by quality control. We used PLINK (version v1.90) [58] to filter out raw SNP data based on the missing rate (mind: 0.01 and geno: 0.01), allele frequency (-maf 0.01), and p values of the Hardy-Weinberg exact test (-hwe 10 − 6). Finally, we obtained a total of 679,920 SNPs that were used for the subsequent population genetic analysis.

**Reference datasets and integration of data**.

We merged genome-wide SNP data from CJZ and CJD with previously reported population data from modern and ancient populations included in the AADR and publicly available genomic projects [5, 25, 26]. It was compiled into two datasets with different SNP densities for the subsequent analysis. We first merged our data with the extracted genotype data of 670 K SNPs in 929 geographically, linguistically, and culturally diverse whole-sequencing genomes from 54 worldwide populations [25] and 317 whole-sequencing genomes in 20 populations from South China, Philippines and Oceania (here we called it as the Oceanian genomic resource) [26], which formed the high-density dataset containing 679,920 SNPs (Table S11). We also merged our data with present-day East Asian populations and ancient people included in the Affymetrix Human Origins (HO) panel to generate the merged HO dataset (Table S12). Data merging was done through EIGENSOFT [59].

### Principal component analysis

We performed a PCA using the smartpca program of EIGENSOFT v.6.1.4 [59] based on the merged data from the worldwide and regional populations from the two datasets with the default parameters. Given the strong linkage disequilibrium of SNPs, we used PLINK [58] to prune them with the parameters “-indep-pairwise 200 25 0.4” before analyzing the PCA. PCA was first performed based on Eastern Eurasian modern populations to explore genetic similarities between East Asian people and Oceania populations. Second, East Asian modern populations were extracted for further intra-regional PCA, in which the ancient samples were projected on the PCs established based on the genetic variations of the modern East Asian people. Focused on the PCA constructed based on the genetic variations of modern and ancient East Asians, we used the basal default parameters and lsqproject: YES.

### Model-based ADMIXTURE analysis

We used the unsupervised model-based statistical technique to dissect the ancestral composition of CJZ and CJD populations. We performed two model-based clustering analyses using ADMIXTURE based on the merged HO and WGS datasets. We used PLINK v1.90 to prune the linked SNPs with the following parameters (r^2^ > 0.4 and --indep-pairwise 200 25 0.4) [60]. After eliminating SNPs with strong linkage disequilibrium, we performed unsupervised ADMIXTURE under 10-fold cross-validation and 100 randomization runs with the predefined numbers of ancestral populations based on global populations and East Asian populations respectively ranging from 2 to 15 and 2 to 10. We used an unsupervised mixing method and calculated the allele frequency of the unmixed ancestry population during the analysis. We terminated the block relaxation algorithm when the objective function δ < 0.0001. We choose the best run and best-fit K value based on log-likelihood and cross-validation error. Ultimately, we observed that the lowest cross-validation errors were K = 13 and K = 2 for the global and East Asian population-based clustering analysis, respectively.

### Fine-scale genetic structure based on FineSTRUCTURE

We used SHAPEIT software (Segmented HAPlotype Estimation & Imputation Tool) to phase our dense SNP data with the default parameters (--burn 10 --prune 10 --main 30) [61]. And we ran ChromoPainterv2 software [62] to paint the target TK and sampled northern and southern East Asians using all-phased populations. To identify the fine-scale population substructure, we conducted fineSTRUCTURE (version 4.0) [62] among modern populations. The Perl scripts of convertrecfile.pl and impute2chromopainter.pl were used to prepare the input phase and recombination data. fineSTRUCTURE, ChromoCombine, and ChromoPainter were used to combine in the four successive steps of analyses with the parameters (-s3iters 100,000 -s4iters 50,000 -s1minsnps 1000 -s1indfrac 0.1).

### Pairwise fst genetic distance, IBD and TreeMix analyses

To explore the possible exiting phylogenetic topologies, we used TreeMix version 1.12 [63] with migration events varying from 0 to 7 to construct a maximum likelihood-based phylogenetic relationship and evaluate the allele frequency distribution of the two studied populations. We calculated the pairwise Fst genetic distance to measure the genetic relationship between CJZ and CJD and other modern populations in East Asia following the method of Weir and Cockerham [64]. Pairwise sharing IBD segments were calculated using Refined-IBD software (16May19. ad5. jar) with the length parameter as 0.1 [65].

### Three/four population tests

We conducted a series of *f*_3_/*f*_4_ statistics using the qp3Pop and qpDstat programs of ADMIXTOOLS [66] with default parameters. We calculated the outgroup-*f*_3_(X, studied TK populations; Mbuti) to measure the shared drift between populations X and studied TK people since their divergence from the outgroup. We used the African Mbuti as the outgroup population. And then, we performed admixture-*f*_3_(X, Y; studied TK populations) to evaluate the potential admixture signature with different ancestral source surrogates. The estimated negative Z-scores less than − 3 indicated that our targeted population was a mixed population with two ancestral sources related to source1 and source2. We computed the *f*_*4*_(W, X; Y, Mbuti) with different reference populations to formally test whether W or X harboured more Y-related ancestry. We applied the *f*_4_-statistics in the form of *f*_4_(TK-speaking1, TK-speaking2; Reference population, Mbuti) to estimate the genetic homogeneity and heterogeneity between studied TK populations and used *f*_4_(Worldwide populations, Dai; TK, Mbuti) to evaluate whether TK people shared alleles with Dai compared with East Asian and non-East Asian populations. Finally, we calculated the *f*_4_(East Asians, TK-speaking; HM/AN/ST, Mbuti) to test whether additional gene flow entered the studied TK populations compared to the used East Asian ancestral source proximity.

### Admixture models and admixture time estimations

We used qpAdm [67] with the default parameters to formally estimate the admixture proportion with predefined northern and southern ancestral East Asian populations. The model was accepted: p-value > 0.05, nested p-value < 0.05 and admixture proportions estimated between (0, 1). We also calculated the admixture times using MALDER [68] with all possible ancestral source pairs. Among the two-way admixture model, we used ancient northern East Asian-related ancestry (Dadiwan_MN) and ancient southern East Asian-related ancestry (Atayal_Taiwan) as sources. Three populations (Laos_Hoabinhian, AR33K, and Tarim_EMBA1) were used as additional outgroups. Seven populations were used as the basic outgroup populations, including Mbuti.SDG, Iran_GanjDareh_N, Italy_North_Villabruna_HG, Mixe.DG, Papuan.DG, Onge.DG and Agta_Philippines. We further used Shimao_LN, Dushan and Paiwan_Taiwan as proxies for ancient Northern East Asian, ancient Southern East Asian, and ancient Southeast Asian-related ancestries in a three-way admixture model. Following the default parameters, GLOBETROTTER was utilized to further identify and date the admixture events based on the shared haplotypes [62].

### Selection sweep analysis

We applied the R package of REHH [69] to run the iHS [70] and the XPEHH [71] tests for capturing haplotype homozygosity-based signals of positive selection. The iHS searches for haplotype structure differences between two alleles in a variant, while the XPEHH approach detects nearly fixed selective sweeps comparing haplotypes in pairs of populations. The XPEHH test was applied to population pairs comparing studied TK populations with northern Eastern Asian populations. The XPEHH score is directional: a positive score suggests that selection will likely happen in studied TK people, whereas a negative score indicates the same about reference populations. The calculation of Fst values between TK populations was performed according to the statistical method of Weir and Cockerham [64], and the top 1000 of the Fst values were selected as the threshold, and the SNP loci above the threshold line were defined as the top selected loci. We combined the resulting loci screened via the three methods to perfume enrichment analysis.

### Functional annotation and pathway enrichment analysis

To explore the biological processes and signaling pathways in which candidate genes may be involved, we performed functional annotation and pathway enrichment analysis using the online tool Metascape [72]. We integrated genes with XPEHH and iHS values variants in the top 1% and top1000 of the Fst as the input gene set, including GO-BP, KEGG, and Reactome Gene Sets, WikiPathways and Canonical Pathways. The top 20 functional categories with -log10 (P value) ≥ 2 were displayed as rich terms and displayed in the enrichment graph. Meanwhile, the relationship between the enriched terms was shown as a network graph, and edges connected the terms with similarity > 0.3.

## Electronic supplementary material

Below is the link to the electronic supplementary material.

Supplementary Material 1

Supplementary Material 2

Supplementary Material 3

Supplementary Material 4

Supplementary Material 5

Supplementary Material 6

Supplementary Material 7

Supplementary Material 8

Supplementary Material 9

Supplementary Material 10

Supplementary Material 11

Supplementary Material 12

Supplementary Material 13

Supplementary Material 14

Supplementary Material 15

Supplementary Material 16

Supplementary Material 17

## Data Availability

The Genome-wide variation data were collected from the public dataset of Allen Ancient DNA Resource (AADR) (https://reich.hms.harvard.edu/allen-ancient-dna-resource-aadr-downloadable-genotypes-present-day-and-ancient-dna-data). The new-generated allele frequency data has been submitted to the public database (https://zenodo.org/record/7233402#.Y8Om-HZBwQ8).
