# Supplementary material for: Extensive genetic admixture between Tai-Kadai-speaking people and their neighbours in the northeastern region of the Yungui Plateau inferred from genome-wide variations
Source: BMC Genomics. 2023 Jun 12;24:317. doi: 10.1186/s12864-023-09412-3 (PMC10259048; doi:10.1186/s12864-023-09412-3)
Supplement: Supplementary file 14 — Supplementary Material 14 [file 12864_2023_9412_MOESM14_ESM.pdf]

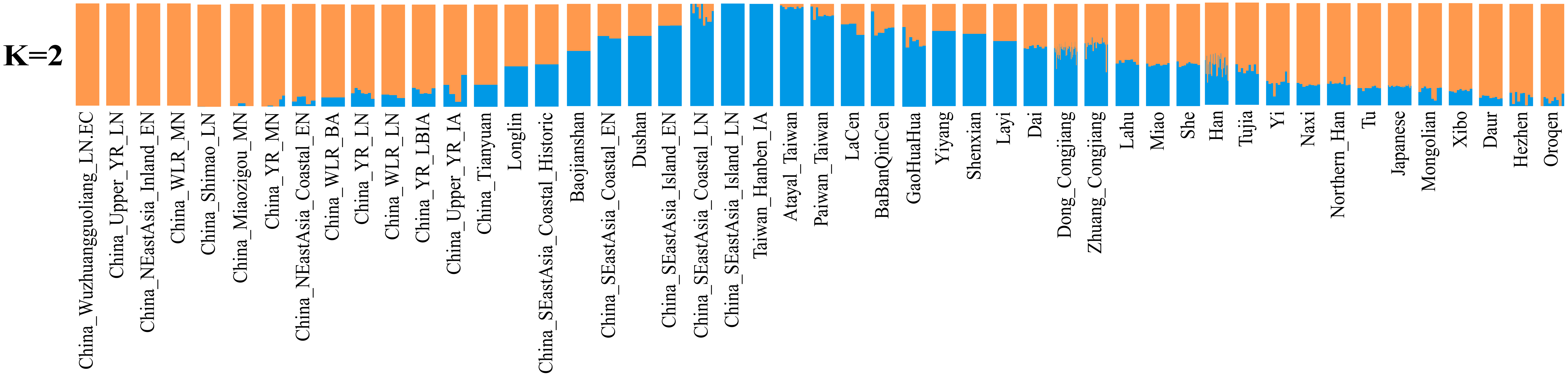

Figure S2. Results of model-based ADMIXTURE clustering analysis among East Asians. Here, we can identify Dong_Congjiang and Zhuang_Congjiang-dominant ancestry maximized in late Neolithic Southern China population and Iron Age Taiwan Hanben. Orange-dominated ancestral components are widely distributed in northern populations.
