# Supplementary material for: Extensive genetic admixture between Tai-Kadai-speaking people and their neighbours in the northeastern region of the Yungui Plateau inferred from genome-wide variations
Source: BMC Genomics. 2023 Jun 12;24:317. doi: 10.1186/s12864-023-09412-3 (PMC10259048; doi:10.1186/s12864-023-09412-3)
Supplement: Supplementary file 15 — Supplementary Material 15 [file 12864_2023_9412_MOESM15_ESM.pdf]

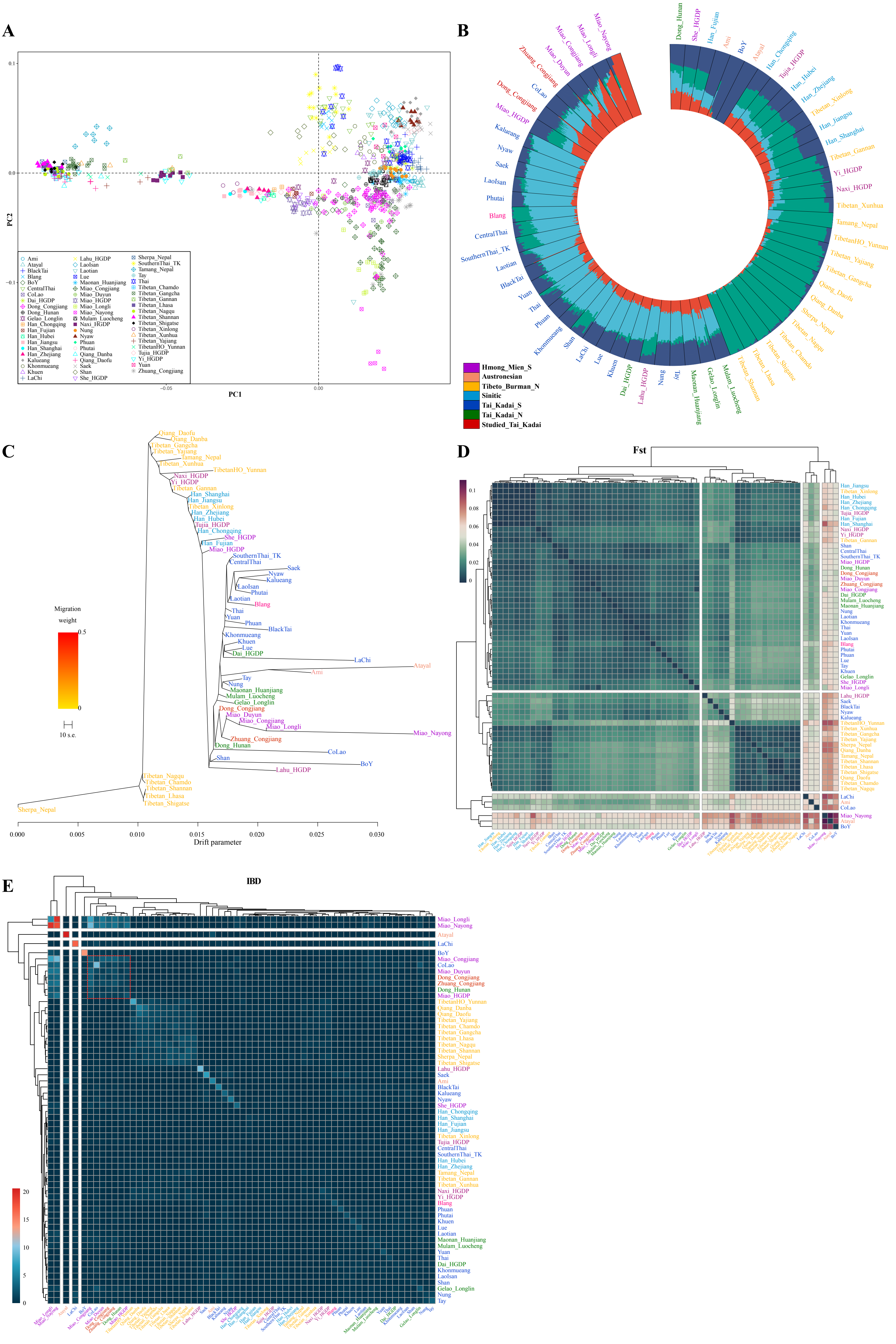

Figure S3. Population substructure of TK speakers and their neighbors in Guizhou aand other regions of China and Southeast Asia. (A) PCA of Guizhou TK people and reference TK people from geographically different South China and Mainland Southeast Asia. (B) Results of model-based ADMIXTURE clustering analysis based on TK, ST, HM, AN and AA speakers. Clustering patterns were visualized with the predefined ancestral sources at K = 4. (C) TreeMix result of haplotype-analyzed datasets. (D) The heatmap of pairwise Fst genetic distance among TK, ST, HM, AN and AA speakers. (E) Pairwise coincidence based on the co-ancestry coefficient.
