# Supplementary figures and images for: Extensive genetic admixture between Tai-Kadai-speaking people and their neighbours in the northeastern region of the Yungui Plateau inferred from genome-wide variations
Source: BMC Genomics. 2023 Jun 12;24:317. doi: 10.1186/s12864-023-09412-3 (PMC10259048; doi:10.1186/s12864-023-09412-3)

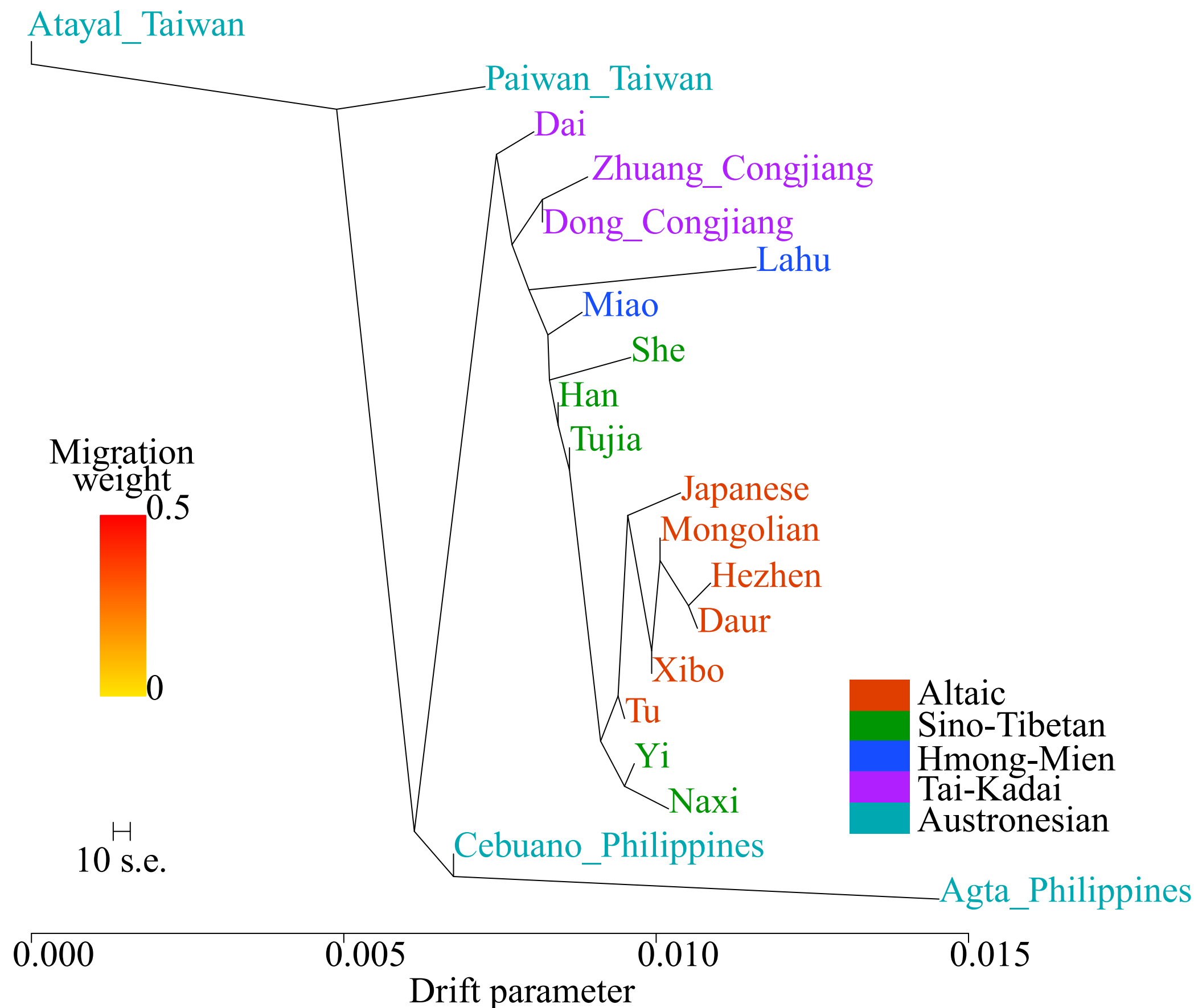

**Figure S4. TreeMix based on the modern Eastern Eurasians.**

Supplement: Supplementary file 16 — Supplementary Material 16 [file 12864_2023_9412_MOESM16_ESM.pdf]
