# Supplementary material for: Extensive genetic admixture between Tai-Kadai-speaking people and their neighbours in the northeastern region of the Yungui Plateau inferred from genome-wide variations
Source: BMC Genomics. 2023 Jun 12;24:317. doi: 10.1186/s12864-023-09412-3 (PMC10259048; doi:10.1186/s12864-023-09412-3)
Supplement: Supplementary file 17 — Supplementary Material 17 [file 12864_2023_9412_MOESM17_ESM.pdf]

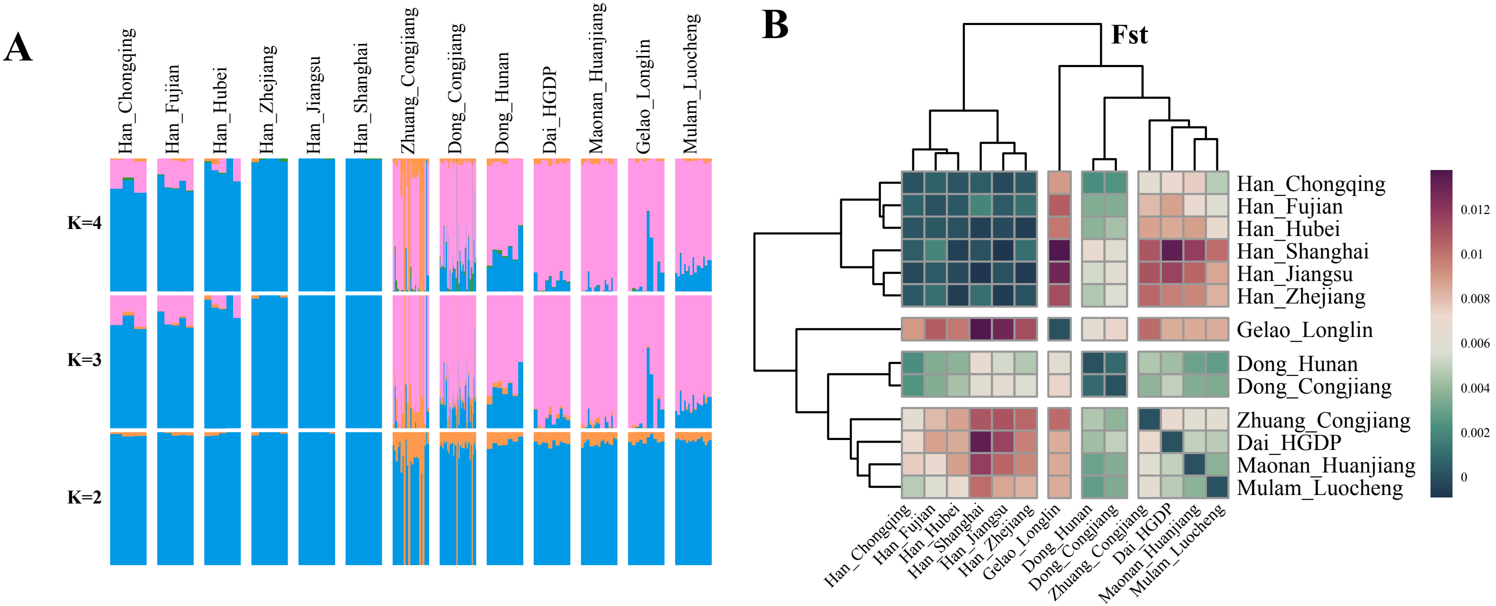

Figure S5. ADMIXTURE model and pairwise Fst genetic distance based on Hans populations and TK populations.(A) Results of model-based ADMIXTURE clustering analysis based on ST speakers and TK speakers. Clustering patterns were visualized with the predefined ancestral sources from K = 2 to K = 4, where the cross-validation error of the K = 2 model was the lowest. (B) Pairwise Fst genetic distance among 6 ST speakers and 7 TK speakers.
