# Supplementary material for: Extensive genetic admixture between Tai-Kadai-speaking people and their neighbours in the northeastern region of the Yungui Plateau inferred from genome-wide variations
Source: BMC Genomics. 2023 Jun 12;24:317. doi: 10.1186/s12864-023-09412-3 (PMC10259048; doi:10.1186/s12864-023-09412-3)
Supplement: Supplementary file 13 — Supplementary Material 13 [file 12864_2023_9412_MOESM13_ESM.pdf]

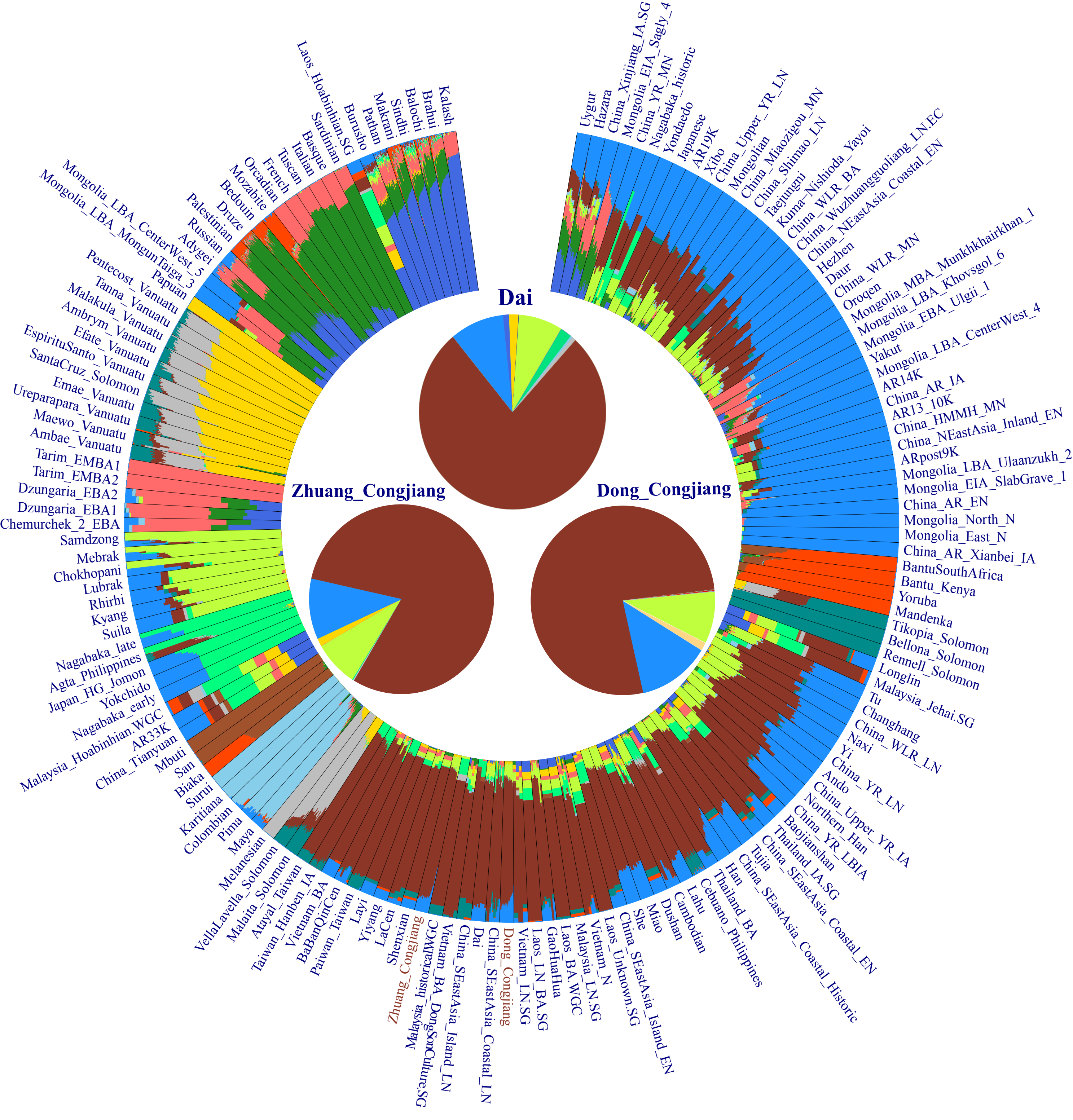

Figure S1. Results of model-based ADMIXTURE clustering analysis based on the merged 670K dataset. Clustering patterns were visualized with the predefined ancestral sources at K = 13. All of these ancestral components were revealed in different colors.
